# Nickel exchanged supported 12-tungstophosphoric acid: synthesis, characterization and base free one-pot oxidative esterification of aldehyde and alcohol†

**DOI:** 10.1039/c8ra08419j

**Published:** 2019-01-11

**Authors:** Anish Patel, Anjali Patel

**Affiliations:** Polyoxometalates and Catalysis Laboratory, Department of Chemistry, Faculty of Science, The Maharaja Sayajirao University of Baroda Vadodara India anjali.patel-chem@msubaroda.ac.in

## Abstract

The present work describes for the first time, the synthesis and characterization of a bi-functional catalyst consisting of nickel and supported 12-tungstophosphoric acid as well as its application in one pot oxidative esterification of benzaldehyde and benzyl alcohol to benzoate ester. The said reactions were operated without any base at a low temperature and atmospheric pressure. The influence of reaction parameters such as nickel concentration, molar ratio of substrate to H_2_O_2_ as well as methanol, catalyst amount, reaction temperature and reaction time were investigated to optimize the conditions for maximum conversion with good selectivity towards the desired product. The superiority of the present work lies in obtaining higher conversion as well as higher selectivity of the desired product with a high TON for both the systems under sustainable reaction conditions. Moreover, the catalyst could be recovered and reused for up to three cycles without any significant loss in its selectivity. The obtained conversion as well as selectivity were discussed with a number of control experiments and based on the obtained results, mechanisms for both the reactions were proposed. Furthermore, the difference in activity towards oxidative esterification of benzaldehyde and benzyl alcohol was also correlated with the proposed mechanism.

## Abstract 2

The synthesis and characterization of a bi-functional catalyst consisting of nickel and supported 12-tungstophosphoric acid are reported as well as its application in one pot oxidative esterification of benzaldehyde and benzyl alcohol to benzoate ester.

## Graphical abstract

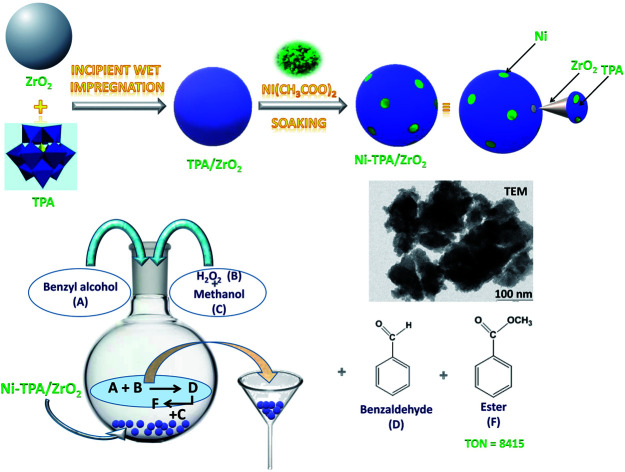

## Introduction

Oxidative esterification of aldehydes with alcohols has received increasing attention during recent years because such raw materials are abundantly available in industry.^1^ Various groups have reported one step conventional methods for oxidative esterification of aldehydes, based on TS-1,^2^ V_2_O_5_-SPC/SPB (SPC-sodium percarbonate, SPB sodium perborate),^3^ supported gold nanoparticle Au/TiO_2_,^4^ Pb and Mg doping in Al_2_O_3_-supported Pd,^5^ palladium/NHC,^6^ manganese phthalocyanine immobilized on silica gel,^7^ supported gold–nickel oxide nanoparticles (AuNiO_*x*_),^8^ magnesia supported gold nanoparticles (Au/MgO),^9^ titanium superoxide,^1^ palladium complex immobilized on magnetite–graphene oxide nanocomposite,^10^ graphite oxide and oxone,^11^ iron oxalate capped iron–copper nanomaterial (Fe(ox)Fe–CuOx),^12^ reduced graphene oxide supported Co_3_O_4_ nanoparticles (Co_3_O_4_/rGO),^13^ supported iron oxide nano particles (FeNP),^14^ gold nanoparticles supported on Ce–Zr oxide,^15^ polystyrene supported rhodium nanoparticles (Rh@PS),^16^ PTFE supported gold nanoparticles^17^ and sulfate radical redox systems.^18^ Many methods suffer from disadvantages such as use of expensive and polluting reagents, an inert atmosphere and lengthy reaction times.^7^

Oxidative esterification of alcohols is highly desirable from both economic and environmental points of view, as alcohols are more readily available as bulk chemicals, more stable than the carbonyl compounds, cheaper, less toxic and easier to handle.^19^ Hence in this direction, to improve the conversion and selectivity of the oxidative esterification of benzyl alcohol reaction, tremendous efforts have been made to develop new catalysts, including supported-monometallic catalysts such as Au, Pt, Pd, Ag, *etc.* and supported-bimetallic catalysts such as Au–Pd, Au–Cu, and Au–Ir catalysts.^20^ Various groups have reported the same, based on Co_3_O_4_–N@C,^21^ palladium complex immobilized on magnetite–graphene oxide nanocomposite,^10^ gold–palladium alloy nanoparticles on a phosphate-modified hydrotalcite support,^22^ supported iron oxide nanoparticles,^14^ Co nanoparticles embedded in nitrogen doped graphite,^23^ polystyrene stabilized rhodium nanoparticles^16^ and Pd–Bi–Te/C.^24^ On the other hand, gold catalysts have considerable interest due to their unique catalytic properties and selectivity in particular reactions^9^ such as Au/TiO_2_,^25^ Au/β-Ga_2_O_3_,^26^ Au nanoparticles on porous coordination polymers,^27^ Au/SiO_2_,^28^ nanocrystalline gold supported on Fe–Ti–Ce-modified mesoporous silica,^29^ Au/ZrO_2_,^30^ Au nanoparticles supported in the nanocages of SBA-16 ^31^ magnesia-supported Au nanoparticles,^9^ Au/Al_2_O_3_ ^32^ and iron doped graphene (Fe-Gr) supported gold.^20^

However, in most cases, liquid base additives (KOH and K_2_CO_3_) are required to obtain a higher yield of the ester.^9^ It would be interesting if the reaction could be carried out using inexpensive metal as well as without base.

A literature survey shows that even though, polyoxometalates based catalysts are found to be excellent, sustainable and have applications for number of oxidation as well as esterification reactions, very few reports are available for the oxidative esterification of the benzaldehyde. To name, anchored 12-tungstophosphoric acid and lacunary anchored phosphotungstate (PW_12_/ZrO_2_, PW_12_/MCM-41, PW_11_/ZrO_2_ and PW_11_/MCM-41),^33^ 12-tungstophosphoric acid immobilized on the surface of silica encapsulated γ-Fe_2_O_3_ nanoparticles^34^ and imidazolium polyoxometalate, [bmim]_3_[PW_12_O_40_].^35^ At the same time, it is very surprising that not a single report is available on oxidative esterification of benzyl alcohol using polyoxometalates.

Recently our group has reported one pot oxidative esterification of benzaldehyde over two catalysts based on lacunary phosphotungstate (i) Cs salt of mono nickel substituted phosphotungstate (CsPW_11_Ni) in homogeneous medium^36^ (ii) supported Cs-salt of mono nickel substituted phosphotungstate catalyst (CsPW_11_Ni/ZrO_2_).^37^ The obtained excellent results, especially in supported nickel catalyst, encourage us to design another catalyst based on nickel and phosphotungstate and extend the work. To achieve same, new bi-functional catalyst was designed by taking the advantage of available protons of supported 12-tungstophosphoric acid (parent phosphotungstate), characterized and evaluated for one pot oxidative esterification of benzaldehyde as well as benzyl alcohol using H_2_O_2_ and methanol. Consequently, the various reaction parameters such as nickel concentration, molar ratio of substrate to H_2_O_2_ as well as methanol, catalyst amount, reaction temperature and reaction time were optimized for the maximum conversion as well as selectivity of the desired product. The catalyst was also recycled and regenerated up to three cycles. Reaction mechanisms for oxidative esterification of benzaldehyde as well as for benzyl alcohol were also proposed and based on this the difference in activity for the reactions were also discussed.

## Experimental

### Materials

All chemicals used were of A. R. grade. Zirconium oxychloride (ZrOCl_2_·8H_2_O) (Loba Chemie), 12-tungstophosphoric acid (H_3_PW_12_O_40_), nickel acetate, benzaldehyde, benzylalcohol, methanol, 30% hydrogen peroxide and dichloromethane obtained from Merck were used as received.

### Catalyst synthesis

#### Synthesis of zirconia supported 12-tungstophosphoric acid (TPA/ZrO_2_)

30% of 12-tungstophosphoric (TPA) was supported on hydrous zirconia (ZrO_2_)^38^ by incipient wet impregnation method as reported by our group earlier.^39^ 1 g of ZrO_2_ was impregnated with aqueous solution of TPA (0.3/30 g mL^−1^ of double distilled water) and dried at 100 °C for 10 h. The obtained material was designated as TPA/ZrO_2_.

#### Synthesis of nickel exchanged supported 12-tungstophosphoric acid

A series of nickel exchanged supported 12-tungstophosphoric acid was synthesized by soaking 1 g of TPA/ZrO_2_ with 25 mL of 0.03–0.05 M solution of nickel acetate for 24 h with stirring. The solution was filtered, removed solid was washed with distilled water in order to remove the excess of nickel and then dried in air at room temperature. The obtained resulting materials were designated as (0.03 M) Ni-TPA/ZrO_2_, (0.04 M) Ni-TPA/ZrO_2_ and (0.05 M) Ni-TPA/ZrO_2_ respectively.

**Scheme 1:**
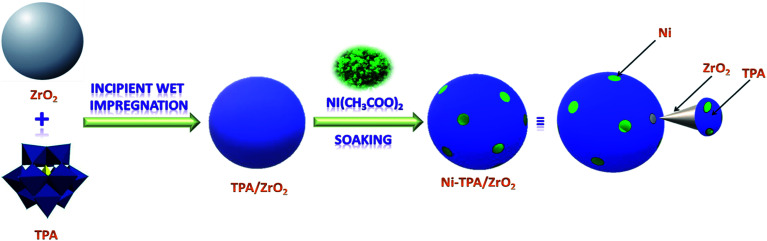
Synthesis of Ni-TPA/ZrO_2_.

The synthesis of Nickel exchanged supported 12-tungstophosphoric acid is shown in Scheme 1.

### Characterization

The amount of nickel was determined by difference in the standard solution as well as sample solution by volumetric method.^40^ The acidity of the catalysts was determined by *n*-butyl amine as well as by potentiometric titration. Thermo gravimetric analysis (TGA) was performed using Mettler Toledo Star SW 7.01 up to 500 °C. Adsorption–desorption isotherms were done through Micromeritics ASAP 2010 surface area analyzer at −196 °C. Specific surface area was calculated using Brunauer–Emmett–Teller (BET method). FT-IR spectrum of the material was performed by using the KBr wafer on a Shimadzu instrument (IRAffinity-1S). The Fourier Transform Raman (FT-Raman) spectra were recorded on a FT-Raman Spectrophotometer (Model Bruker FRA 106). Electron spin resonance (ESR) spectra were recorded on a Varian E-line. Century series X-band ESR spectrometer at liquid nitrogen temperature and scanned from 2500 to 5000 gauss. X-ray photoelectron spectroscopy (XPS) measurements were performed with Auger Electron Spectroscopy (AES) Module PHI 5000 Versa Prob II. Powder X-ray Diffraction (Powder XRD) was carried out using Philips Diffractometer (Model PW-1830). TEM analysis was carried out on JEOL (JAPAN) TEM instrument (model-JEM 100CX II) with accelerating voltage of 200 kV. The samples were dispersed in ethanol and ultrasonicated for 5–10 min. A small drop of the sample was then taken in a carbon coated copper grid and dried before viewing.

### Acidity measurement

#### Total acidity by *n*-butyl amine titration

A 0.025 M solution of *n*-butyl amine in toluene was prepared for assessment of total acidity of the catalyst.^41^ The 0.25 g catalyst was suspended in 0.025 M *n*-butyl amine solution for 24 h and the excess base was titrated against trichloroacetic acid in toluene using neutral red as an indicator.

#### Potentiometric titration

The type of acidic sites as well as acidic strength was investigated by employing potentiometric titration with 0.05 N *n*-butylamine which helps in computing different acid sites.^42^ 0.5 g of catalyst sample was suspended in 50 mL acetonitrile and the mixture was aged at 25 °C. 0.05 N *n*-butyl amine in acetonitrile solution was added in equal time periods and the potential (mV) was recorded.

### Catalytic evaluation

Oxidative esterification reaction of benzaldehyde/benzylalcohol (10 mmol) with H_2_O_2_ (30 mmol), methanol (5/7.5 mL respectively) and catalyst (10 mg) were charged in a 50 mL batch reactor provided with a double walled air condenser, magnetic stirrer, and a guard tube. The reaction mixture was refluxed at 80 °C for 6 h/24 h respectively. In both the cases, the obtained products were extracted with dichloromethane and analyzed on a gas chromatograph (Shimadzu-2014) using a capillary column (RTX-5).

## Results and discussion

### Characterization of catalyst

The volumetric analysis of nickel in standard solution and filtrate show the presence of 0.24 wt%, 0.3 wt% and 0.4 wt% of Ni in (0.03 M) Ni-TPA/ZrO_2_, (0.04 M) Ni-TPA/ZrO_2_, and (0.05 M) Ni-TPA/ZrO_2_ respectively. Here, low exchanged amount of nickel in each catalyst, indicates that only the protons of TPA were exchanged by the Ni. EDX value of W (16.45 wt%) and Ni (0.32 wt%) in (0.04 M) Ni-TPA/ZrO_2_ is in good agreement with calculated one (17.17 wt% of W, 0.30 wt% of Ni). Very low % of Ni indicates that only the protons of TPA were exchanged.

The types and strength of the acidic sites were determined by potentiometric titration. The strength of acidic sites in terms of initial electrode potential is shown in Table 1. The drastic increase in acidic sites as well as acidic strength of all nickel-based catalysts compare to that of TPA/ZrO_2_, may be due to the exchange of available protons of TPA/ZrO_2_ by nickel. It is interesting to note down that, as increase in nickel concentration, the total numbers of acidic sites are also increases and this may be due to the Lewis acidity of Ni. The total acidity of (0.05 M) Ni-TPA/ZrO_2_ is higher compare to (0.04 M) Ni-TPA/ZrO_2_ but the acidic strength and strong acidic sites are less, this may be due to blocking of strong acidic sites at higher exchange. Hence, (0.04 M) Ni-TPA/ZrO_2_ was selected for detail characterization and re-designated as Ni-TPA/ZrO_2_.

**Table 1:** Potentiometric titration

Catalyst	Acidic strength Ei (mV)	Types of acidic sites (meq. g^−1^)		Total no. of acidic sites
		Strong	Weak	
ZrO_2_	18	0.1	2.1	2.2
TPA/ZrO_2_	45	1.5	1.2	2.7
(0.03 M) Ni-TPA/ZrO_2_	61	1.9	1.4	3.3
(0.04 M) Ni-TPA/ZrO_2_	87	2.3	1.7	4.0
(0.05 M) Ni-TPA/ZrO_2_	70	2.1	2.1	4.2

TGA curve (Fig. S1†) of TPA/ZrO_2_ shows 12.6% weight loss in the temperature range of 70–100 °C indicating the loss of adsorbed water molecules. Further, there was no weight loss observed up to 500 °C indicating the stability of the supported catalyst. TGA of Ni-TPA/ZrO_2_ shows initial weight loss of 8.5% up to 180 °C indicating the loss of adsorbed water molecules. Besides this, also no significant weight loss was observed up to 500 °C, suggests higher thermal stability of synthesized catalyst.

**Fig. 1:**
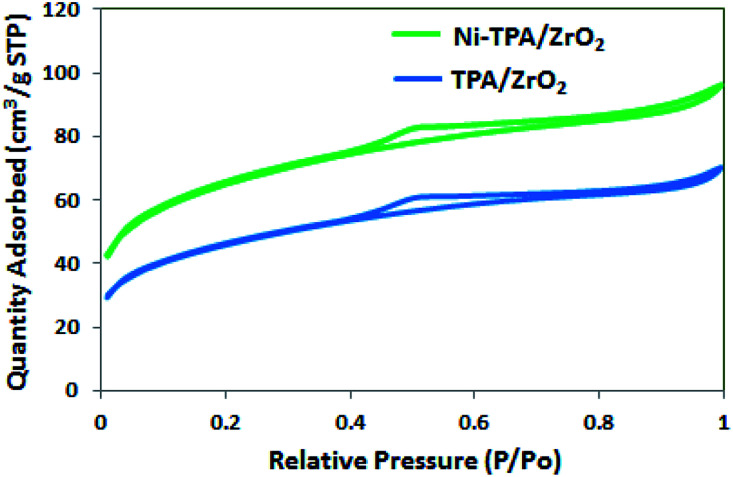
Nitrogen adsorption desorption isotherms.

The BET surface area of ZrO_2_, TPA/ZrO_2_ and Ni-TPA/ZrO_2_ were measured. Specific surface area decreased for supported catalysts TPA/ZrO_2_ (146 m^2^ g^−1^) as compared to that of support ZrO_2_ (170 m^2^ g^−1^) and in good agreement with reported fact^43^ that there may be decrease in surface area in case of the supported catalyst in which oxides are used as supports. This is because of strong interaction of TPA with the oxide support. The surface area of Ni-TPA/ZrO_2_ (221 m^2^ g^−1^) is higher as compare to that of TPA/ZrO_2_, indicating the exchange of available surface protons of the TPA/ZrO_2_ with Ni as well as high dispersion of Ni on the surface of TPA/ZrO_2_, which is also seen in nitrogen adsorption desorption isotherms (Fig. 1). It also confirms no change in the basic structure.

**Fig. 2:**
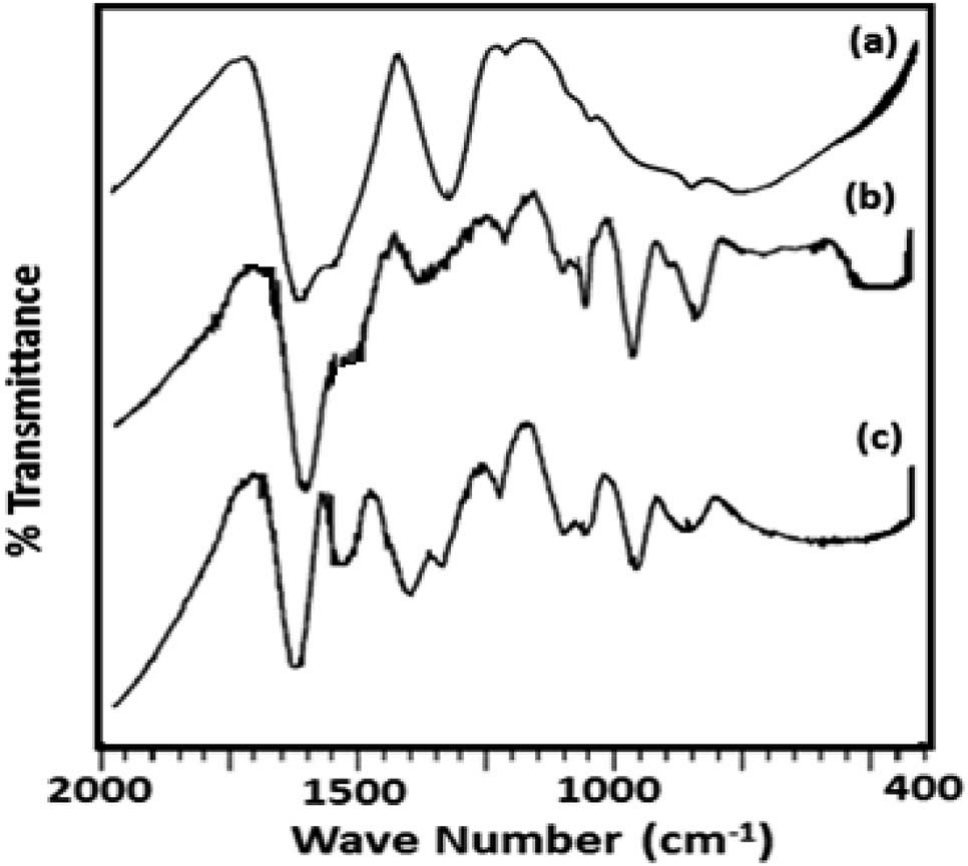
FT-IR spectra of (a) ZrO_2_ (b) TPA/ZrO_2_ and (c) Ni-TPA/ZrO_2_.

The FT-IR spectra of ZrO_2_ (Fig. 2) shows broad bands in the region of 3400, 1600 and 1370, and 600 cm^−1^ attributed to O–H asymmetric stretches, H–O–H and O–H–O bending, and Zr–OH bending respectively.^38^ FT-IR spectrum of TPA/ZrO_2_ (Table S1†) exhibits bands at 1070; 964; and 812 cm^−1^ corresponding to P–O, W=O, and W–O–W stretching vibration frequencies respectively. The FT-IR spectrum of Ni-TPA/ZrO_2_ also shows all the characteristic bands for TPA but with significant shift which may be due to the exchange of Ni with available protons of TPA, with an additional band at 495 cm^−1^ is observed attributing to Ni–O vibration.

Raman spectra of ZrO_2_ (Fig. S2†) shows broad peaks in the region from 200 to 800 cm^−1^, which are associated with long-range disordering arrangement in amorphous state.^44^ Raman spectra of TPA/ZrO_2_ shows bands at 1010, 990, 900, 550, and 217 cm^−1^, which are assigned to *υ*_s_ (W–O_d_), *υ*_as_ (W–O_d_), *υ*_as_ (W–O_b_–W), *υ*_s_ (W–O_c_–W), and *υ*_s_ (W–O_a_), respectively where O_a_, O_b_, O_c_, and O_d_ correspond to the oxygen atoms linked to phosphorus, to oxygen atoms bridging two tungsten (from two different triads for O_b_ and from the same triad for O_c_), and to the terminal oxygen W=O, respectively. Raman spectra of Ni-TPA/ZrO_2_ shows bands at 947, 929, 859, 532 and 232, corresponding to *υ*_s_ (W–O_d_), *υ*_as_ (W–O_d_), *υ*_as_ (W–O_b_–W), *υ*_s_ (W–O_c_–W), and *υ*_s_ (W–O_a_) respectively. The decrease in intensities of the bands as well as significant shifting^45^ in all the characteristic bands may be due to change in the environment because of the exchange of surface protons of TPA by nickel.

The full range (5000–2500 G) X-band liquid nitrogen temperature ESR spectrum for Ni-TPA/ZrO_2_ (Fig. S3†) was recorded. The calculated *g* value (*g* ∼ 2.05) confirms the presence of Ni(ii) species, in good agreement with reported one.^46^ Further, the presence of Ni(ii) is confirmed by XPS.

**Fig. 3:**
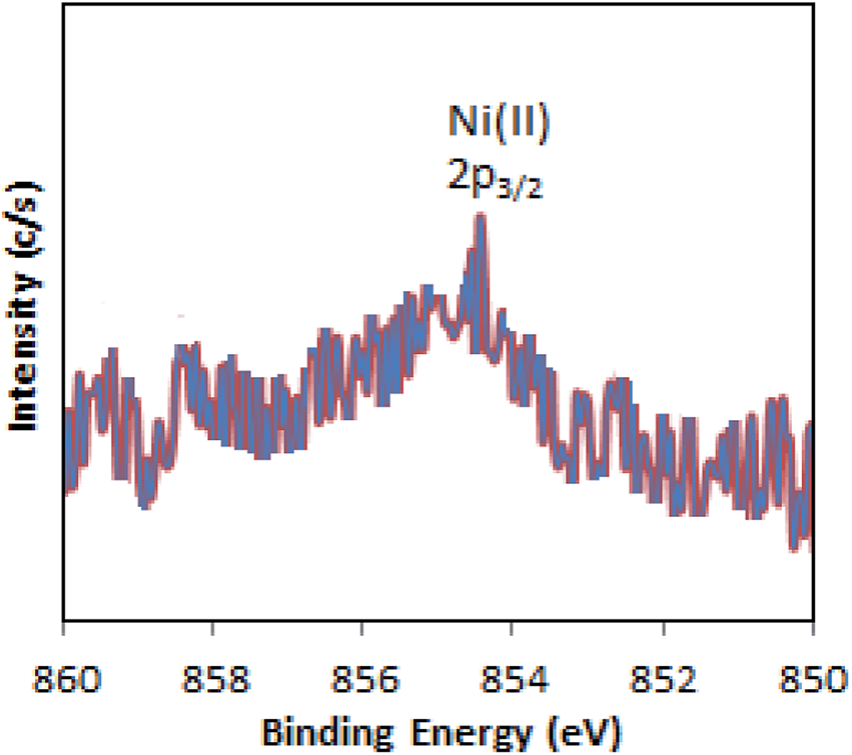
X-ray photoelectron spectrum of Ni-TPA/ZrO_2_.

The XPS of Ni-TPA/ZrO_2_ is displayed in Fig. 3. A low intense peak at 854.5 eV (2p_3/2_) is in good agreement with reported one^47^ confirming the presence of Ni(ii) on the surface. However, the obtained poor-quality spectra with low peak intensity of 2p_3/2_ may be due to the presence of very low concentration of Ni (0.3 %wt) in the synthesized catalyst.

**Fig. 4:**
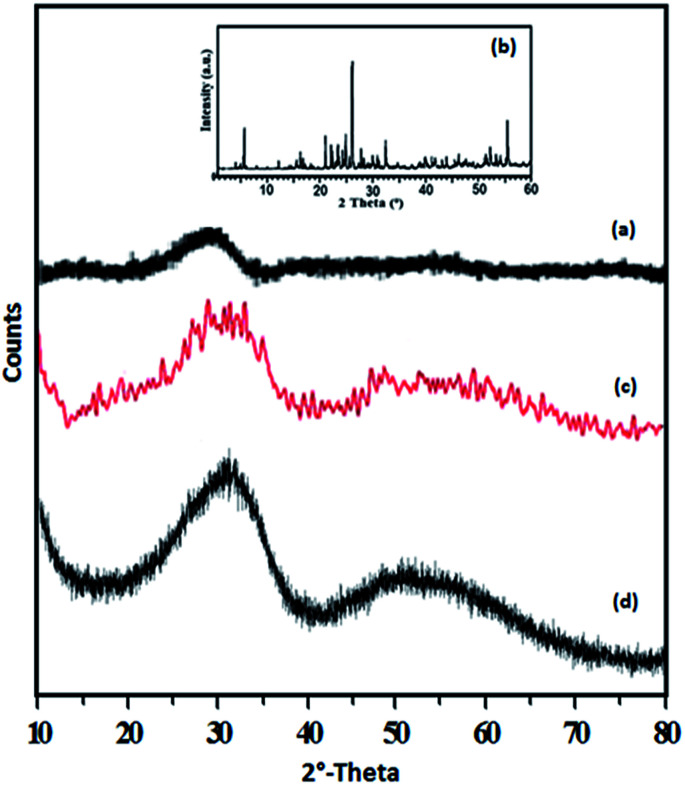
XRD spectra of (a) ZrO_2_, (b) TPA (c) TPA/ZrO_2_ and (d) Ni-TPA/ZrO_2_.

The XRD patterns of ZrO_2_, TPA, TPA/ZrO_2_ and Ni-TPA/ZrO_2_ catalysts are shown in Fig. 4. The XRD patterns of ZrO_2_ (Fig. 4a) shows the amorphous nature of the support. Absence of characteristic diffraction lines corresponding to TPA, in the XRD pattern of TPA/ZrO_2_ (Fig. 4c), indicates a high dispersion of TPA on the surface of ZrO_2_. The XRD patterns of Ni-TPA/ZrO_2_ (Fig. 4d) are very similar to TPA/ZrO_2_, suggesting very high dispersion of nickel on the non-crystalline surface of TPA/ZrO_2_, which is also observed in TEM.

TEM images of the fresh catalyst Ni-TPA/ZrO_2_ are presented in (Fig. S4†) at various magnifications. SAED image (a) indicates the non-crystalline and uniform distribution of nickel in the synthesized catalyst. Images (b and c) show the uniform dispersion of Ni on the surface of TPA/ZrO_2_.

In summary, FT-IR and Raman spectrum indicate the retention of TPA structure in the catalyst as well as exchange of nickel with available surface protons of TPA/ZrO_2_. ESR and XPS studies confirm the presence of Ni(ii) in the catalyst. While XRD and TEM reveals the uniform dispersion of Ni over TPA/ZrO_2_.

### Catalytic activity

#### Oxidative esterification of benzaldehyde

**Scheme 2:**
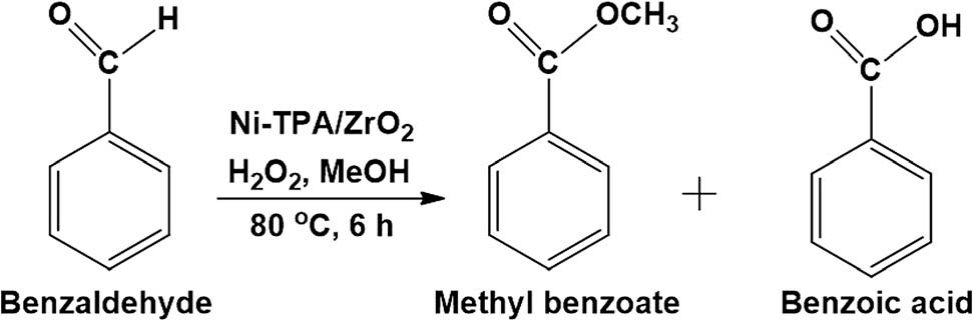
Oxidative esterification of benzaldehyde.

To evaluate the efficiency of the catalyst, for the oxidative esterification of benzaldehyde into their corresponding esters, benzaldehyde and methanol were selected as test substrates in the presence of hydrogen peroxide as an oxidant (Scheme 2). Effect of different reaction parameters such as nickel concentration, molar ratio of substrate to H_2_O_2_ as well as methanol, catalyst amount, reaction temperature and reaction time were studied to optimize the conditions for maximum conversion.

The reaction was carried out in presence of different concentration of nickel and the results are presented in Table S2.† It can be seen that initially, % conversion increases with increase in nickel concentration without change in % selectivity of the desired product. This can be explained on the bases of the acidity of the catalyst (Table 1). With increase in concentration of nickel, the % conversion also increases due to the increase in total number of acidic sites of the catalyst. With further increase in nickel concentration (0.05 M) the % conversion decreases with increase in % selectivity of the desired product. This may be due to the lower acidic strength of the (0.05 M) Ni-TPA/ZrO_2_ compare to Ni-TPA/ZrO_2_ (Table 1). Conversion was achieved highest in case of Ni-TPA/ZrO_2_ compared to (0.03 M) Ni-TPA/ZrO_2_ and (0.05 M) Ni-TPA/ZrO_2_. Hence, further optimization was carried out using Ni-TPA/ZrO_2_.

**Fig. 5:**
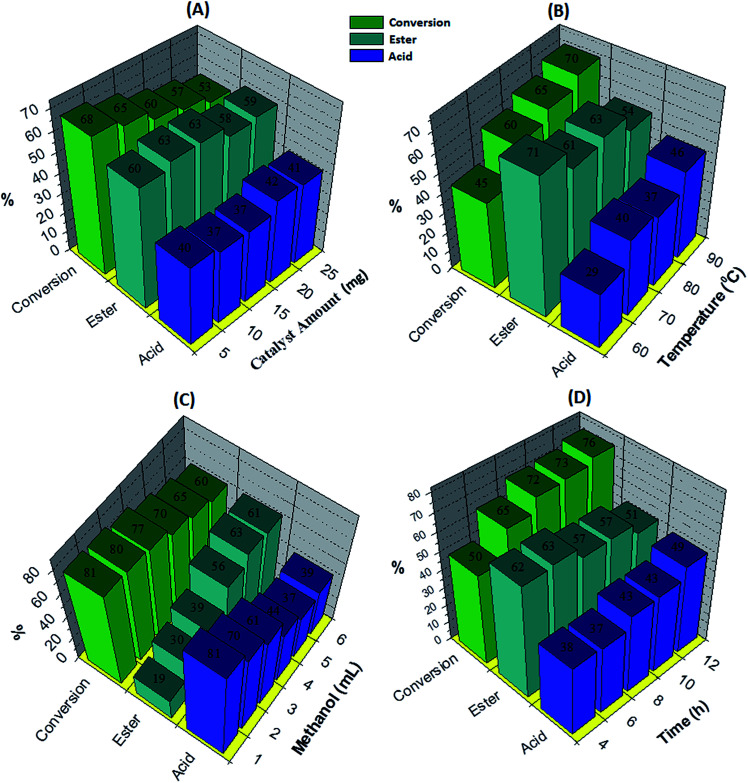
Optimization of parameters for oxidative esterification of benzaldehyde. Optimization of parameters for oxidative esterification of benzaldehyde. Reaction conditions: (A) effect of the catalyst amount: benzaldehyde (10 mmol), H_2_O_2_ (30 mmol), methanol (5 mL), catalyst/substrate ratio (5.11 × 10^−5^), time (6 h), temp. (80 °C); (B) effect of the temperature: benzaldehyde (10 mmol), H_2_O_2_ (30 mmol), methanol (5 mL), catalyst (10 mg), catalyst/substrate ratio (5.11 × 10^−5^), time (6 h); (C) effect of the methanol amount: benzaldehyde (10 mmol), H_2_O_2_ (30 mmol), catalyst (10 mg), catalyst/substrate ratio (5.11 × 10^−5^), time (6 h), temp. (80 °C) and (D) effect of the time: benzaldehyde (10 mmol), H_2_O_2_ (30 mmol), methanol (5 mL), catalyst/substrate ratio (5.11 × 10^−5^), temp. (80 °C).

The effect of the catalyst amount was evaluated by varying it from 5 to 25 mg (Fig. 5A). By increasing the catalyst amount up to 10 mg conversion was decreases with increase in % selectivity of desired product, with further increase in catalyst amount, % conversion as well as selectivity decreases. This is due to unproductive decomposition of H_2_O_2_ which generates additional water and results in decrease in selectivity of ester, is in good agreement with the reported one.^37^ Maximum % conversion with highest % selectivity of ester was obtained for 10 mg of catalyst. The temperature effect screened from 60 to 90 °C, with increase in temperature conversion also increases with decrease in ester selectivity (Fig. 5B), may be due to the thermal decomposition of H_2_O_2_.^37^ 80 °C temperature was optimized to achieve maximum conversion as well as ester selectivity. Then, effect of methanol volume was carried out from 1 to 6 mL (Fig. 5C). Results show that with enlarging methanol amount, conversion decreases with increase in ester selectivity. Here, decrease in conversion is because of dilution of the substrate concentration.^37^ From obtained results, 5 mL of methanol was optimized. Finally, the time effect was performed with in 4 h to 12 h range (Fig. 5D). Results indicate that with rise in time the conversion increases whereas selectivity of ester decreases. This is because of decomposition of H_2_O_2_ which results in the formation of the water molecule to hydrolyze the ester to aldehyde. From the results, 6 h time was optimized for the reaction. The obtained results are summarized in Fig. 5.

Further to see the effect of H_2_O_2_, the reaction was carried out with different mole ratio of substrate : H_2_O_2_ keeping all other parameters constant (Table 2). From the results it can be seen that the conversion increases with increasing oxidant amount up to mole ratio (1 : 3) and is in good agreement with the chemical dynamics^34^ according to which the oxidative esterification could be improved by increasing the amount of H_2_O_2_. Simultaneously, the selectivity of the desired product, ester decreases while that of acid increases because higher amount of oxidant will tolerate more benzaldehyde to oxidize in benzoic acid. Further with increase in oxidant amount (1 : 4) the drastic decrease in conversion was found and hence substrate to oxidant ratio (1 : 3) was optimized.

**Table 2:** Effect of mole ratio^a^

Substrate : H_2_O_2_	% Conversion	% Selectivity	
		Ester	Acid
1 : 1	34	74	26
1 : 2	54	70	30
1 : 3	65	63	37
1 : 4	51	56	44

a Reaction conditions: benzaldehyde (10 mmol), oxidant (H_2_O_2_), methanol (5 mL), catalyst (10 mg), time (6 h), temp. (80 °C).

From the above study, the conditions optimized for the maximum conversion are: benzaldehyde (10 mmol), H_2_O_2_ (30 mmol), methanol (5 mL), conc. of Ni (5.11 × 10^−4^ mmol), catalyst/substrate ratio (5.11 × 10^−5^), time (6 h), temp. (80 °C). Conversion = 65% with TON = 12 712.

#### Control experiments and investigation of mechanism

Number of experiments were carried out in order to study the role of each substrate (Table S3†) as well as catalyst: (i) without catalyst (benzaldehyde + methanol + H_2_O_2_) the reaction gives negligible conversion (ii) without H_2_O_2_ (benzaldehyde + methanol + catalyst) no significant reaction occurs, (iii) without methanol (benzaldehyde + H_2_O_2_ + catalyst), oxidation of aldehyde to benzoic acid takes place. In all three cases, benzoic acid was obtained as a single product. This study shows that both H_2_O_2_ and methanol as well as catalyst are essential for the oxidative esterification reaction to take place.

The control experiments were also carried out with Ni(CH_3_COO)_2_, ZrO_2_, TPA/ZrO_2_ and Ni-TPA/ZrO_2_ in identical experimental conditions and results are presented in Table 3. ZrO_2_ and TPA/ZrO_2_ both were moderately active with low selectivity of the desired product benzoate ester. However, Ni(CH_3_COO)_2_ gives 54% conversion and 52% ester selectivity as expected. The increase in the conversion as well as selectivity in case of Ni-TPA/ZrO_2_ shows the synergic effect due to the presence of nickel, which is also confirmed by acidity measurement. Total number of acidic sites as well as acidic strength (Table 1) is almost double and responsible for increase in activity.

**Table 3:** Control experiments for oxidative esterification of benzaldehyde^a^

Catalyst	% Conversion	% Selectivity	
		Ester	Acid
^a^Ni(CH_3_COO)_2_	54	52	48
ZrO_2_	27	41	59
TPA/ZrO_2_	52	43	57
^a^Ni-TPA/ZrO_2_	65	63	37

a Reaction conditions: benzaldehyde (10 mmol), H_2_O_2_ (30 mmol), methanol (5 mL), catalyst (10 mg), catalyst/substrate ratio (5.11 × 10^−5^), time (6 h), temp. (80 °C). ^a^Active amount of nickel (5.11 × 10^−4^ mmol).

**Scheme 3:**
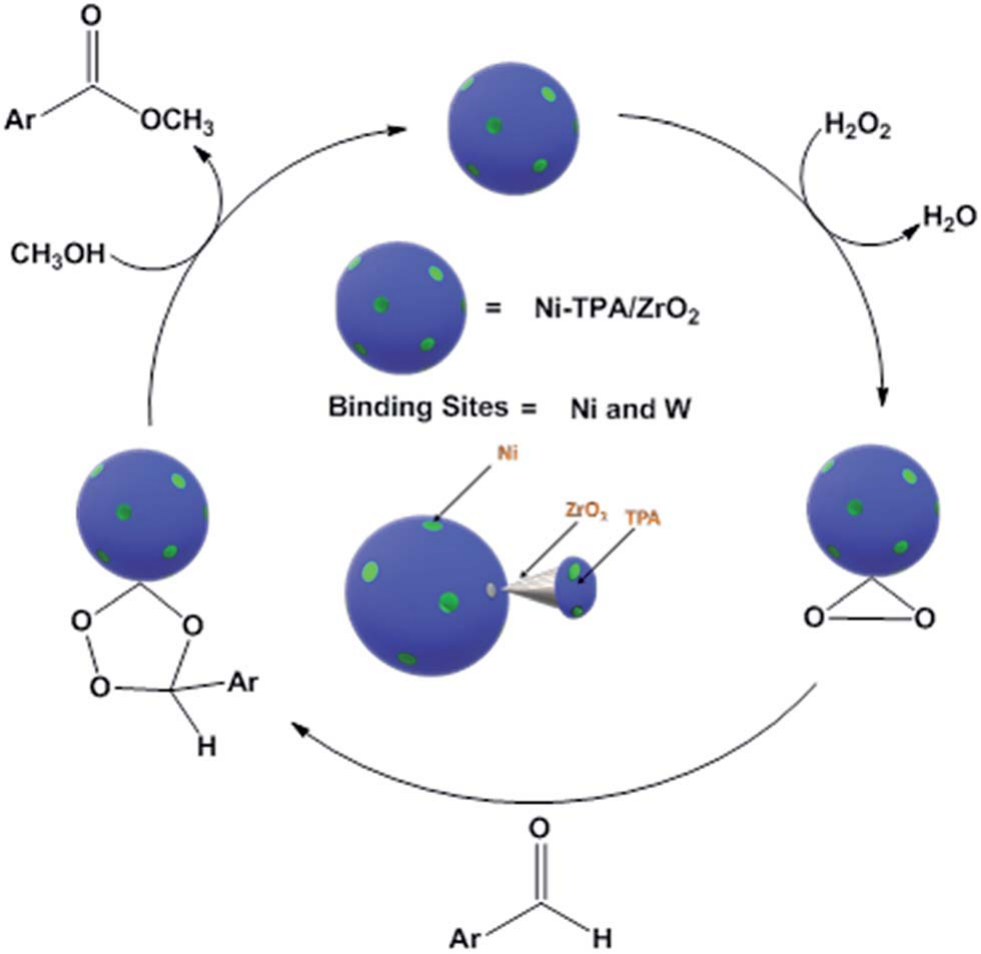
Proposed reaction mechanism for oxidative esterification of benzaldehyde.

For oxidative esterification of benzaldehyde, we are expecting the same mechanism as reported by us earlier,^36^ contradictory to general mechanism. The general mechanism demonstrates the formation of acetal as intermediate.^48^ To explain the contradiction, following set of experiments was carried out. (1) When benzoic acid is used as a substrate instead of benzaldehyde under identical conditions (in absence of H_2_O_2_), 34% conversion with 100% selectivity of methyl benzoate was obtained. (2) In another reaction, benzaldehyde was reacted with methanol in presence of catalyst and absence of H_2_O_2_ gives 11% conversion with 100% selectivity for benzoic acid in 6 h. On prolonging the reaction for 3 h after addition of H_2_O_2_, 46% conversion was achieved with 60% ester selectivity. The study shows that the oxidation of benzaldehyde does not proceed through an acetal intermediate, but through *in situ* formation of benzoic acid. The proposed reaction mechanism is shown in Scheme 3. Benzaldehyde is first converted into benzoic acid in presence of H_2_O_2_ and the formed benzoic acid is esterified in presence of methanol to desire ester. It must be noted that both the steps take place *in situ*. Here, it might be possible that in the beginning, the formation of metal-peroxo active intermediate (here, metal can be both, W as well as Ni) takes place.^49^ However, as reported by us earlier, the detail mechanism of the synthesis of esters involving POM and transition metal is not clear.^49^

#### Oxidative esterification of benzyl alcohol

**Scheme 4:**
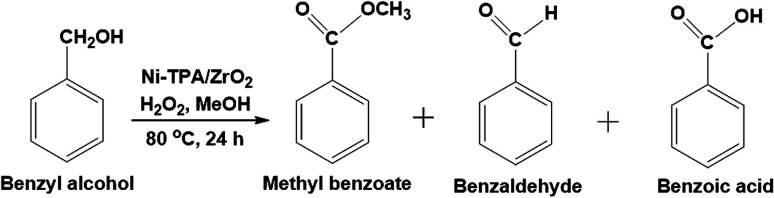
Oxidative esterification of benzyl alcohol.

To evaluate the competence of the catalyst, for the direct conversion of into their corresponding esters, benzyl alcohol and methanol were selected as test substrates in the presence of hydrogen peroxide as an oxidant (Scheme 4). The influence of different reaction parameters such as nickel concentration, molar ratio of substrate to H_2_O_2_ as well as methanol, catalyst amount, reaction temperature and reaction time was studied to optimize the conditions for maximum conversion.

To determine the effect of nickel concentration, the reaction was carried using (0.03 M) Ni-TPA/ZrO_2_, Ni-TPA/ZrO_2_ and (0.05 M) Ni-TPA/ZrO_2_. The obtained results are shown in Table S4.† The trend in the % conversion as well as % selectivity was found to be the same as earlier (Table S2†). And hence, further optimization was carried out using Ni-TPA/ZrO_2_.

**Fig. 6:**
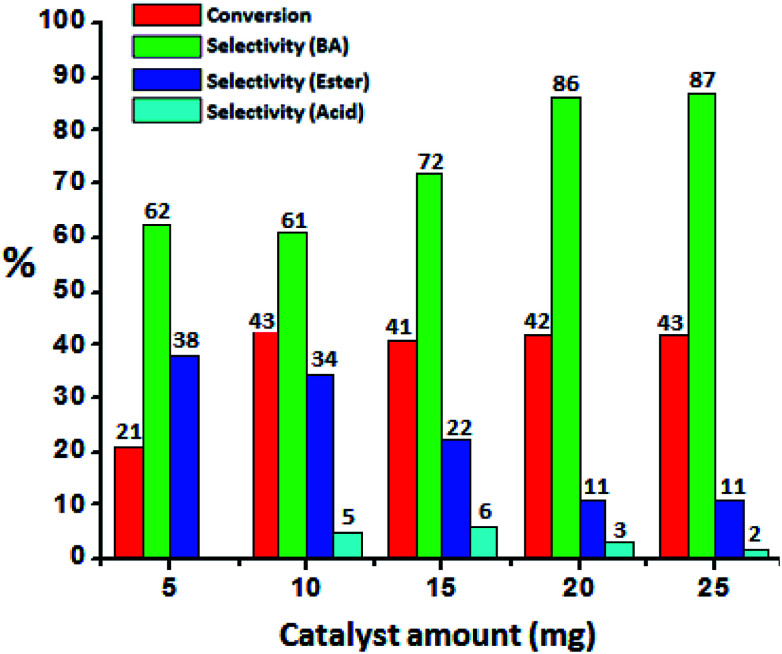
Effect of catalyst amount. Reaction conditions: benzyl alcohol (10 mmol), H_2_O_2_ (30 mmol), methanol (7.5 mL), time (24 h), temp. (80 °C).

To optimize the catalyst amount, the reaction was performed by varying amount (*i.e.* from 5 to 25 mg) by keeping other parameters constant. Oxidative esterification is significantly affected by acidity as well as oxidizing property of the catalyst. The increase in the conversion can be attributed to an increase in the number of available catalytically active sites. From Fig. 6 it can be seen that initially the conversion increases with increasing the amount from 5 to 10 mg. Further, with increasing the amount, the conversion as well as selectivity of ester decreases as observed earlier (Fig. 5A). This may be due to the rapid catalytic decomposition of H_2_O_2_ in presence of excess amount of catalyst which generates the water molecules.^37^ As esterification is reversible process in presence of water, the selectivity of the ester product decreases with increase in catalyst amount.

Effect of methanol amount was studied in the range of 1 mL to 8 mL and obtained results are presented in Table 4. Initially, the amount of methanol increased up to 7.5 mL, the % conversion remains almost same with increase in the % selectivity of the desired product. With further increase in methanol quantity (8 mL), % conversion decreases; this is because of higher the volume of methanol, which dilute the concentration of the substrates. Hence, 7.5 mL volume of methanol was optimized for the reaction for further study.

**Table 4:** Effect of methanol amount^a^

Methanol (mL)	% Conversion	% Selectivity		
		Benzaldehyde	Ester	Acid
1	38	61	13	26
2	45	77	11	12
3	49	75	15	10
4	48	75	16	9
5	47	72	21	7
6	46	69	27	4
7	45	65	31	4
7.5	43	61	34	5
8	35	55	40	5

a Reaction condition: benzyl alcohol (10 mmol), H_2_O_2_ (30 mmol), catalyst (10 mg), catalyst/substrate ratio (5.11 × 10^−5^), temp. (80 °C), time (24 h).

**Fig. 7:**
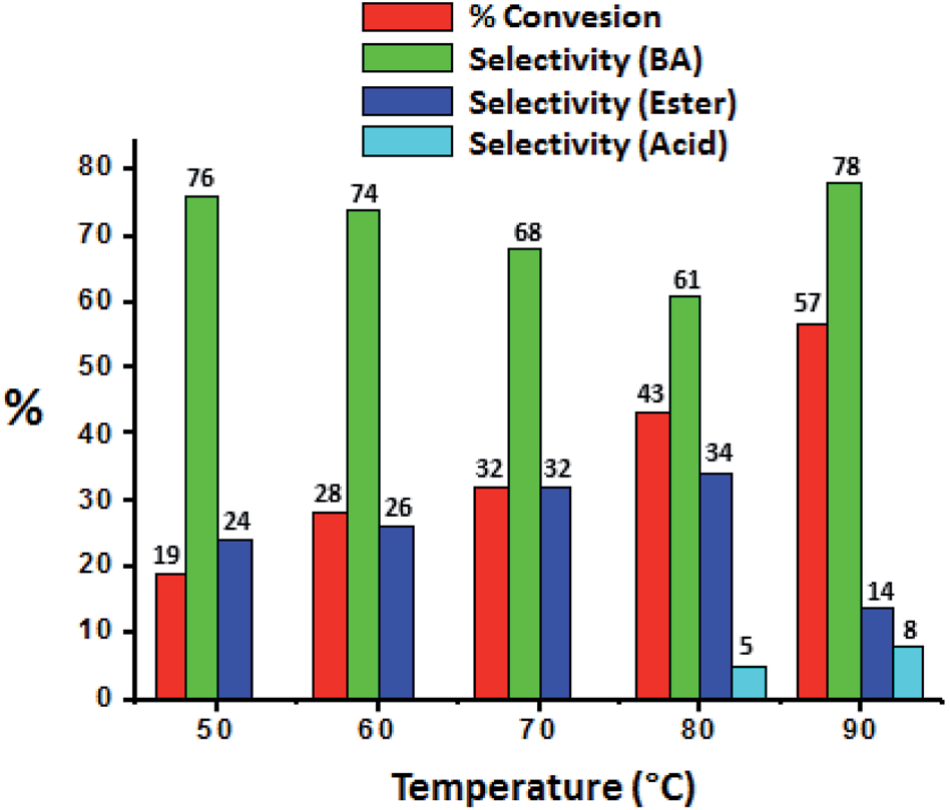
Effect of temperature. Reaction condition: benzyl alcohol (10 mmol), H_2_O_2_ (30 mmol), methanol (7.5 mL), catalyst (10 mg), catalyst/substrate ratio (5.11 × 10^−5^), time (24 h).

The effect of temperature was carried out and obtained results were presented in Fig. 7, it shows that conversion as well selectivity of ester increases with increase in the temperature from 50 °C to 80 °C. Further, with increase in temperature to 90 °C, conversion also increases but ester selectivity decreases. This may be due to the thermal decomposition of the H_2_O_2_. Decomposition of H_2_O_2_ produces water molecule and hence ester hydrolysed to benzaldehyde.^37^

The effect of time was investigated by varying the reaction time. As shown in Fig. S5,† the reaction was performed at three different time intervals. Initially, increase in the reaction time from 18 h to 24 h, conversion as well as ester selectivity increases. Further, increasing the reaction time shows no notable change in the results because of attainment of reaction equilibrium.^37^ So, 24 h is optimized for the maximum reaction conversion and ester selectivity.

Finally, to see the effect of H_2_O_2_, the reaction was carried out with different mole ratio of substrate : H_2_O_2_ keeping all other parameters constant (Table 5). From the results it can be seen that the conversion increases with increasing the oxidant amount with the selectivity of desired product ester up to mole ratio (1 : 3). Because it is well known that the oxidative esterification could be improved by increasing the amount of H_2_O_2_, according to the chemical dynamics.^34^ Further with increase in oxidant amount (1 : 4) the slight decrease in conversion was found. Hence, (1 : 3) substrate to oxidant ratio was optimized for further catalytic optimization.

**Table 5:** Effect of mole ratio^a^

Substrate : H_2_O_2_	% Conversion	% Selectivity		
		Aldehyde	Ester	Acid
1 : 1	10	89	11	—
1 : 2	34	80	20	—
1 : 3	43	61	34	5
1 : 4	41	41	33	4

a Reaction conditions: benzyl alcohol (10 mmol), oxidant (H_2_O_2_), methanol (7.5 mL), catalyst (10 mg), time (24 h), temp. (80 °C).

From the above study, the conditions optimized for the maximum conversion (43%) with TON = 8415 are: benzyl alcohol (10 mmol), H_2_O_2_ (30 mmol), methanol (7.5 mL), conc. of Ni (5.11 × 10^−4^ mmol), catalyst/substrate ratio (5.11 × 10^−5^), time (24 h) and temp. (80 °C).

***Control experiments and investigation of mechanism.*** Number of experiments (Table S5†) were carried out in order to study the role of each substrate as well as catalyst: (i) without catalyst (benzyl alcohol + methanol + H_2_O_2_), the reaction did not progress significantly (ii) without oxidant (benzyl alcohol + methanol + catalyst) reaction did not occur, (iii) without methanol (benzyl alcohol + H_2_O_2_ + catalyst), oxidation of benzyl alcohol to benzaldehyde takes place. Two things can be concluded from the above studies (i) H_2_O_2_, methanol as well as catalyst are essential for the feasibility of the oxidative esterification reaction and (ii) W and Ni both are responsible (due to the synergic effect) for the oxidation as in later case the found conversion was almost double.

The control experiments were also carried out with Ni(CH_3_COO)_2_, ZrO_2_, TPA/ZrO_2_ and Ni-TPA/ZrO_2_ in identical experimental conditions and results are presented in Table 6. ZrO_2_ and TPA/ZrO_2_ both were moderately active with low selectivity of the desired product, benzoate ester. In presence of nickel salt Ni(CH_3_COO)_2_, only 5% conversion with 32% ester selectivity was obtained. The increase in the conversion as well as selectivity in case of Ni-TPA/ZrO_2_ shows the synergic effect due to the presence of nickel, which is also confirmed by acidity measurement. Total number of acidic sites as well as acidic strength (Table 1) is almost double and responsible for increase in activity.

**Table 6:** Control experiments for oxidative esterification of benzyl alcohol^a^

Catalyst	% Conversion	% Selectivity		
		Benzaldehyde	Ester	Acid
^a^Ni(CH_3_COO)_2_	5	68	32	—
ZrO_2_	10	77	23	—
TPA/ZrO_2_	22	61	27	12
^a^Ni-TPA/ZrO_2_	43	61	34	5

a Reaction conditions: benzyl alcohol (10 mmol), H_2_O_2_ (30 mmol), methanol (7.5 mL), catalyst (10 mg), catalyst/substrate ratio (5.11 × 10^−5^), time (24 h), temp. (80 °C). ^a^Active amount of nickel (5.11 × 10^−4^ mmol).

**Scheme 5:**
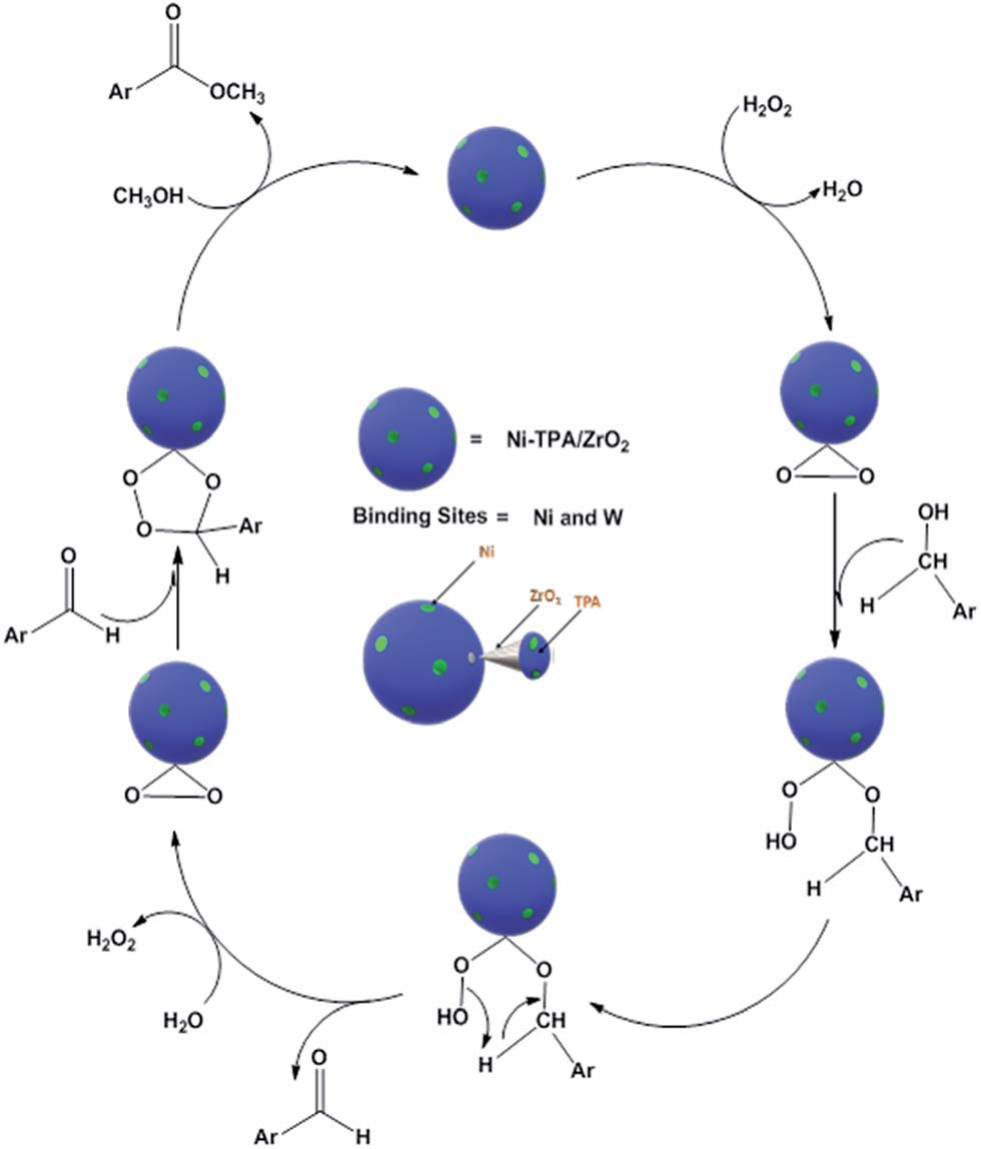
Proposed reaction mechanism for oxidative esterification of benzyl alcohol.

For oxidative esterification of benzyl alcohol, two possible mechanisms are known (1) two step mechanism involving oxidation of alcohol to benzaldehyde and further oxidative esterification of benzaldehyde to ester *via* benzoic acid formation and (2) formation of hemiacetal as an intermediate,^25,26^ whose further dehydration gives the ester. In order to know about the possible mechanism, the following experiments have been carried out. (1) Benzyl alcohol was reacted with H_2_O_2_ in presence of catalyst, without adding methanol, under the identical conditions, 23% conversion with 98% selectivity of benzaldehyde was obtained. Benzoic acid was obtained with poor selectivity (2%). (2) Benzyl alcohol was reacted with methanol in presence of catalyst, without adding H_2_O_2_, no conversion was found. However, on prolonging the same reaction with addition of H_2_O_2_ up to 24 h, 41% conversion was achieved with % selectivity 68, 26 and 8 for benzaldehyde, ester and acid respectively. The obtained results are consistent with two step mechanism *i.e.* oxidation of benzyl alcohol to benzaldehyde (also confirmed from Table S5†) and further oxidative esterification of benzaldehyde to ester formation. Hence, we are assuming same mechanism, as proposed for oxidative esterification of aldehyde. Only the difference is that in present case, first alcohol is converted in to aldehyde and then it follows the same mechanism. Based on the above study, the following reaction mechanism is proposed (Scheme 5).

It is interesting to note that activity of the catalyst for oxidative esterification of benzaldehyde is greater than benzyl alcohol. This is in good agreement with the proposed mechanism, which shows that the latter involves one more additional step. *i.e.* oxidation of benzyl alcohol to benzaldehyde.

### Heterogeneity test

In both the cases, heterogeneity test was carried out by centrifuging the catalyst from the reaction mixture. In the case of oxidative esterification of benzaldehyde (Table 7) reaction was carried out up to 4 h, then reaction mixture was centrifuged and filtrate was allowed to react up to 6 h. Similarly, oxidative esterification of benzyl alcohol (Table 8), reaction was carried out for 12 h, centrifuged to remove the catalyst and then filtrate was allowed to react up to 24 h. After that obtained reaction mixtures were extracted by using dichloromethane and analyzed by Gas chromatogram. Results showed that there was no significant change in conversion of the reaction after removing the catalyst, indicates no leaching of Ni during the reaction. This study indicates that TPA plays an important role to bind Ni very strongly and thus does not allow the leaching of Ni into the reaction mixture, making it a true heterogeneous catalyst to recycle and reuse. Here, heterogeneous nature of the catalyst and withholding of the active species on the support during the reaction shows that present catalyst is of category C.^50^

**Table 7:** Heterogeneity test for oxidative esterification of benzaldehyde reaction^a^

Catalyst	% Conversion	% Selectivity	
		Ester	Acid
Ni-TPA/ZrO_2_	50 (after 4 h)	62	38
	50 (after 6 h)	61	39

a Reaction conditions: benzaldehyde (10 mmol), H_2_O_2_ (30 mmol), methanol (5 mL), catalyst (10 mg), catalyst/substrate ratio (5.11 × 10^−5^), temp. (80 °C).

**Table 8:** Heterogeneity test for oxidative esterification of benzyl alcohol reaction^a^

Catalyst	% Conversion	% Selectivity		
		Benzaldehyde	Ester	Acid
Ni-TPA/ZrO_2_	20 (after 12 h)	71	25	4
	19 (after 24 h)	72	24	4

a Reaction conditions: benzyl alcohol (10 mmol), H_2_O_2_ (30 mmol), catalyst (10 mg), methanol (7.5 mL), catalyst/substrate ratio (5.11 × 10^−5^), temp. (80 °C).

**Fig. 8:**
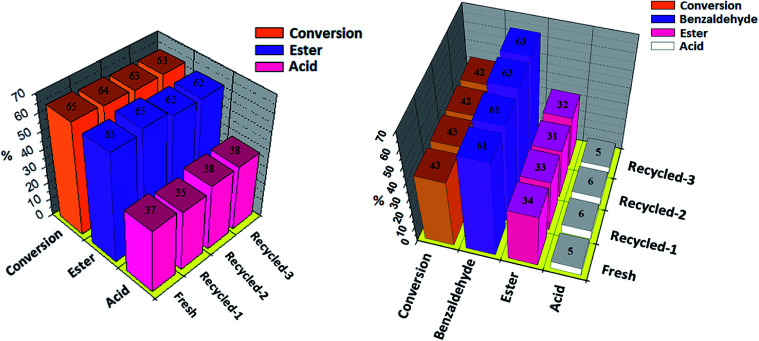
Oxidative esterification of (a) benzaldehyde and (b) benzyl alcohol with fresh and regenerated catalyst. Reaction conditions: benzaldehyde (10 mmol), H_2_O_2_ (30 mmol), methanol (5 mL), time (6 h). Reaction conditions: benzyl alcohol (10 mmol), H_2_O_2_ (30 mmol), methanol (7.5 mL), time (24 h). Catalyst (10 mg), temp. (80 °C), catalyst/substrate ratio (5.11 × 10^−5^).

The recovery and reusability of the catalyst is a key issue for the sustainability of any catalytic process. Results of recyclability for oxidative esterification of benzaldehyde and oxidative esterification of benzyl alcohol are shown in Fig. 8 (a and b) respectively for Ni-TPA/ZrO_2_. After completion of reaction, the organic layer was extracted by dichloromethane. The catalyst was recovered by simple centrifugation, washed with dichloromethane followed by water and then dried in oven at 100 °C for an hour and finally used for the next cycle. Present catalyst was examined up to three cycles only, due to the small scale of reaction with very low concentration of catalyst. In both the cases obtained results show that there was no significant change in conversion as well as in desired product selectivity. The constant results indicate the activity of the catalyst towards its repetitive use and its true heterogeneity nature during the reaction.

### Characterization of regenerated catalyst

Regenerated catalyst was characterized by elemental analysis (EDX), *n*-butyl amine acidity, potentiometric acidity, FT-IR and TEM analysis for the confirmation of the catalyst structure retention.

EDX values of Ni (0.34 wt%) and W (16.52 wt%) of regenerated Ni-TPA/ZrO_2_ is in good agreement with values of fresh catalyst (16.45 wt% of Ni, 0.32 wt% of Ni) confirming no emission of Ni as well as TPA from ZrO_2_ during the reaction.

The acidity and total number of acidic sites of the fresh and regenerated catalyst were determined by volumetric titration with *n*-butyl amine as well as by potentiometric titration, as shown in Table S6† respectively. From the tables it can be seen that acidic sites of regenerated catalyst slightly decrease compared to fresh catalyst whereas acidic strength in terms of initial electrode potential also decreases. This obtained result indicates that few strong acidic sites get blocked during the reaction. Here, blocking of strong acidic sites did not alter the activity of the regenerated catalyst, which can be seen from the catalytic activity of the regenerated catalyst as shown in Fig. 8.

**Fig. 9:**
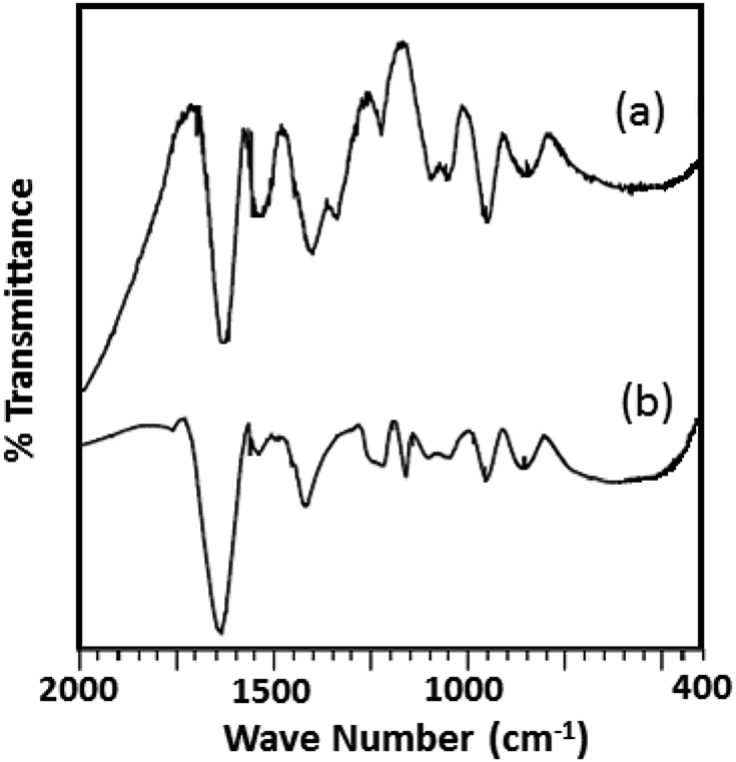
FTIR spectra of (a) Ni-TPA/ZrO_2_ and (b) R-Ni-TPA/ZrO_2_.

The FTIR spectra of the fresh catalyst and regenerated catalyst are shown in Fig. 9. From the figure it can be seen that almost identical spectrum was obtained without any significant shift in the bands of regenerated catalyst (R-Ni-TPA/ZrO_2_) compare to the fresh catalyst (Ni-TPA/ZrO_2_), indicates that catalyst structure remains unaltered even after the regeneration. However, the spectrum is slightly different from the fresh one in terms of intensity. This might be due to the sticking of the substrates on the surface, although this might not be significant in the reutilization of the catalyst.^51^

**Fig. 10:**
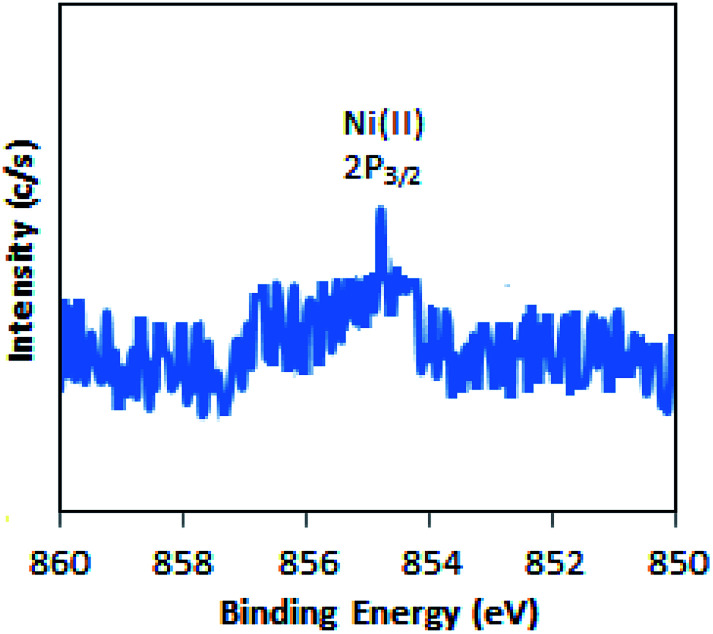
XPS of regenerated Ni-TPA/ZrO_2_.

The X-ray photoelectron spectrum of regenerated Ni-TPA/ZrO_2_ is displayed in Fig. 10. A low intense peak at 854.6 eV (2p_3/2_) is in agreement with reported one^47^ and confirms the presence of Ni(ii) on the surface. Obtained spectrum is identical with the fresh catalyst Ni-TPA/ZrO_2_, confirms that catalyst is sustainable during the reaction.

TEM images of regenerated catalyst Ni-TPA/ZrO_2_ are displayed in Fig. S6† at various magnifications. SAED image (a) indicates the non-crystalline and uniform distribution of nickel in the regenerated catalyst. Images (b and c) show the dark uniform suspension in the amorphous nature of the catalyst. This indicates the uniform dispersion of the catalyst over the surface of the support. Obtained images were identical with the fresh catalyst, show the stability and sustainability of the catalyst during the reaction.

### Comparison with reported catalyst

Catalytic activity of the present catalyst is also compared with reported one (Table 9). Literature survey shows that only two reports are available, and that are by our group only, in which polyoxometalate and nickel based catalysts are used for the oxidative esterification of benzaldehyde: (i) Cs salt of mono nickel substituted phosphotungstate (CsPW_11_Ni) in homogeneous medium^36^ and (ii) heterogeneous catalyst, supported Cs salt of mono nickel substituted phosphotungstate (CsPW_11_Ni/ZrO_2_).^37^ Comparison shows that present catalyst gives higher % conversion compared to CsPW_11_Ni/ZrO_2_, which may be due to presence of higher total number of acidic sites whereas selectivity of ester was low due to lower acidic strength. However, homogeneous catalyst CsPW_11_Ni gives the identical % conversion compare to present catalyst, but it must be noted that in case of homogeneous catalyst the active amount of nickel was 3 times more compared to present catalyst.

**Table 9:** Comparison with reported catalyst^a^

Catalyst	Acidic strength Ei (mV)	Types of acidic sites (meq. g^−1^)			Total no. of acidic sites	% Conversion	% Selectivity	
		Very Strong	Strong	Weak			Ester	Acid
^a^ Ni-TPA/ZrO_2_	87	0	2.3	1.7	4.0	65	63	37
^b^ CsPW_11_Ni^36^	50	0	0.7	1.7	2.4	65	55	45
^c^ CsPW_11_Ni/ZrO_2_ ^37^	110	0.2	1.3	2.2	3.7	40	75	25

a Reaction conditions: benzaldehyde (10 mmol), H_2_O_2_ (30 mmol), methanol (5 mL), catalyst (10 mg), catalyst/substrate ratio (5.11 × 10^−5^), time (6 h), temp. (80 °C). ^a^Active amount of nickel (5.11 × 10^−4^ mmol). ^b^Active amount of nickel (1.5 × 10^−3^ mmol). ^c^Active amount of nickel (5 × 10^−4^ mmol).

At the same time, not a single report is available on oxidative esterification of benzyl alcohol using polyoxometalate.

## Conclusion

New versatile bi-functional heterogeneous catalyst comprising nickel and supported 12-tungstophosphoric acid was introduced for base free one pot oxidative esterification of benzaldehyde as well as benzyl alcohol. The catalyst proved to be a bi-functional one by combining their acidic and redox properties. The superiority of present work lies in obtaining higher conversion as well as higher selectivity of the desired product with high TON (>8000 in both the cases) under sustainable reaction conditions. The activity of the catalyst for oxidative esterification of benzyl alcohol was lower than benzaldehyde because the former reaction involves one more additional step. *i.e.* oxidation of benzyl alcohol to benzaldehyde, which undergoes oxidative esterification. The catalyst can be recycled up to three cycles without any degradation. In addition, the advantages of using Ni as an alternative of more expensive metals as heterogeneous catalyst makes this methodology interesting from an economic as well as an ecological point of view.

## Conflicts of interest

There are no conflicts to declare.

## Supplementary Material

RA-009-C8RA08419J-s001
