# Supplementary material for: Nickel exchanged supported 12-tungstophosphoric acid: synthesis, characterization and base free one-pot oxidative esterification of aldehyde and alcohol
Source: RSC Adv. 2019 Jan 11;9(3):1460–71. doi: 10.1039/c8ra08419j (PMC9059559; doi:10.1039/c8ra08419j)
Supplement: RA-009-C8RA08419J-s001 [file RA-009-C8RA08419J-s001.pdf]

Electronic Supplementary Material (ESI) for RSC Advances.
This journal is © The Royal Society of Chemistry 2018

Supplementary Information

Nickel Exchanged Supported 12-tungstophosphoric acid: Synthesis, Characterization and Base free one pot Oxidative Esterification of Aldehyde and Alcohol

*Anish Patel and Anjali Patel **

Polyoxometalates and Catalysis Laboratory, Department of Chemistry, Faculty of Science.

The Maharaja Sayajirao University of Baroda, Vadodara, India.

*E-mail: anjali.patel-chem@msubaroda.ac.in

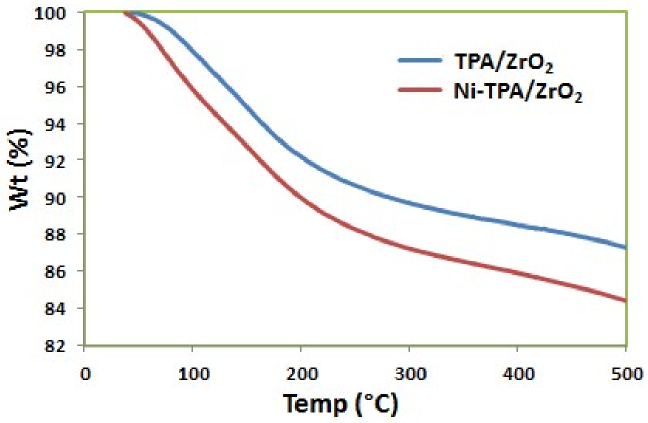

Figure S1. TGA Curves

Table S1. FT-IR data of (a) ZrO_2, (b) TPA/ZrO_2 and (c) Ni-TPA/ZrO_2

Materials	Wavenumber (cm^{-1})						
	O-H-O	H-O-H	P-O	W=O	W-O-W	Zr-O-H	Ni-O
ZrO_2	1600	137	-	-	-	600	-
TPA/ZrO_2	1600	1370	1070	964	812	600	-
Ni-TPA/ZrO_2	1631	1404	1059	947	812	600	495

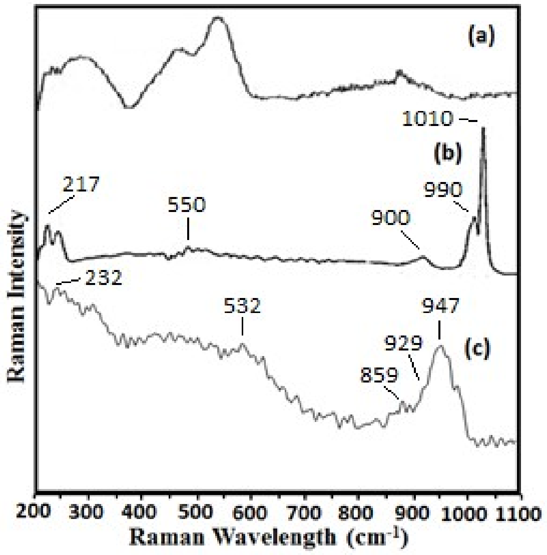

Figure S2. Raman data of (a) ZrO_2, (b) TPA/ZrO_2 and (c) Ni-TPA/ZrO_2

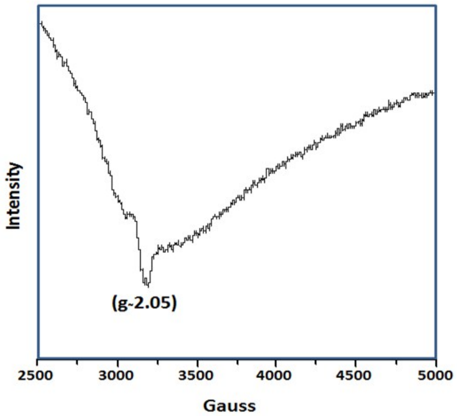

Figure S3. ESR spectrum of Ni-TPA/ZrO_2

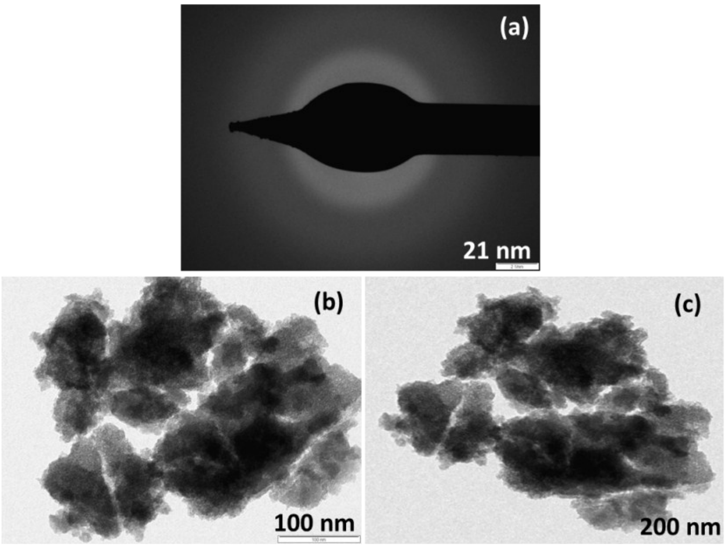

Figure S4. TEM images of dispersed Ni-TPA/ZrO_2

Table S2. Effect of nickel concentration

Catalyst	% Conversion	% Selectivity	
		Ester	Acid
(0.03M) Ni-TPA/ZrO_2	60	64	36
*Ni-TPA/ZrO_2	65	63	37
(0.05M) Ni-TPA/ZrO_2	58	69	31

Reaction conditions: Benzaldehyde (10 mmol), H_2O_2 (30 mmol), Methanol (5 mL), Catalyst (10 mg), Time (6 h), Temp. (80 °C). * Ni-TPA/ZrO_2 = (0.04 M) Ni-TPA/ZrO_2

Table S3. Role of substrate

**Catalyst**	**% Conversion**	**% Selectivity**	
		**Ester**	**Acid**
Without catalyst	3	-	100
Without H_2O_2	11	-	100
Without methanol	42	-	100

Reaction conditions: Benzaldehyde (10 mmol), H_2O_2 (30 mmol), Methanol (5 mL), Catalyst (10 mg), Catalyst/Substrate ratio (5.11e-5), Time (6 h), Temp. (80 °C).

Table S4. Effect of nickel concentration

Catalyst	% Conversion	% Selectivity		
		Benbaldehyde	Ester	Acid
(0.03M) Ni-TPA/ZrO_2	42	68	28	4
*Ni-TPA/ZrO_2	43	61	34	5
(0.05M) Ni-TPA/ZrO_2	59	90	8	2

Reaction conditions: Benzyl alcohol (10 mmol), H_2O_2 (30 mmol), Methanol (7.5 mL), Catalyst (10 mg), Time (24 h), Temp. (80 °C). *Ni-TPA/ZrO_2 = (0.04 M) Ni-TPA/ZrO_2

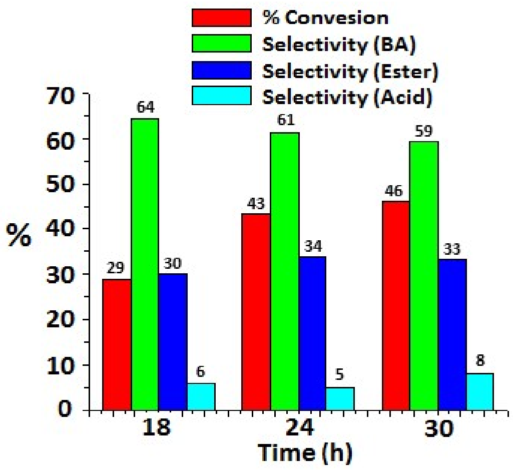

Figure S5. Effect of time. Reaction condition: Benzyl alcohol (10 mmol), H_2O_2 (30 mmol), Methanol (7.5 mL), Catalyst (10 mg), Catalyst/Substrate ratio (5.11e-5), Temp. (80 °C)

Table S5. The roll of substrates and catalyst

Condition	% Conversion	% Selectivity	
		**Benzaldehyde**	**Acid**
Without Catalyst (with methanol + H_2O_2)	-	-	-
Without H_2O_2 (with methanol + catalyst)	0.2	100	-
Without Methanol	[a]1.6 (with $Ni(CH_3COO)_2$)	100	-
	12.7 (with TPA/ZrO_2)	100	-
	23 (with $Ni\text{-}TPA/ZrO_2$)	98	2

Reaction conditions: Benzyl alcohol (10 mmol), H_2O_2 (30 mmol), Methanol (7.5 mL), Catalyst (10 mg), Catalyst/Substrate ratio (5.11e-5), Time (24 h), Temp. (80 °C). [a]Active amount of nickel (5.11×10^{-4} mmol).

Table S6. Acidity by n-butyl amine volumetric titration and Potentiometric titration

Catalyst	Acidic strength Ei (mV)	Types of acidic sites ($meq.g^{-1}$)		Total no. of acidic sites	Acidic sites mmol of n-butyl amine/g
		Strong	Weak		
$Ni\text{-}TPA/ZrO_2$	87	2.3	1.7	4.0	0.86
$R\text{-}Ni\text{-}TPA/ZrO_2$	82	2.1	1.6	3.7	0.82

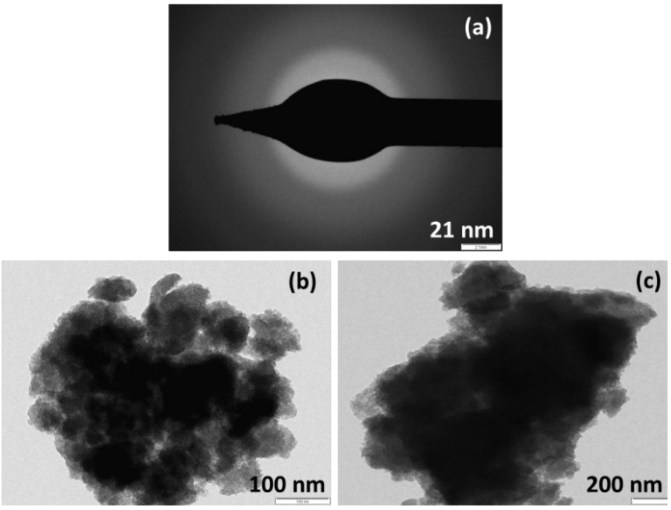

Figure S6 TEM images of regenerated $Ni\text{-}TPA/ZrO_2$
